# Prospective, Open-Label, and Three-Arm Investigator-Initiated Study to Compare the Efficacy and Safety of Three Estradiol Treatment Protocols for Endometrial Preparation in Frozen Embryo Transfer (FET) Cycles

**DOI:** 10.7759/cureus.111081

**Published:** 2026-06-18

**Authors:** Molina Patel, Niket Patel, Nayana Patel, Harsha Bhadarka, Paresh Ghoghari, Sachin Dhaduk, Kairavi Vyas, Dipal Parmar

**Affiliations:** 1 Obstetrics &amp; Gynecology, Akanksha Hospital and Research Institute, Anand, IND; 2 Reproductive Medicine, Akanksha Hospital and Research Institute, Anand, IND; 3 Embryology, Akanksha Hospital and Research Institute, Anand, IND; 4 Clinical Research, Akanksha Hospital and Research Institute, Anand, IND

**Keywords:** assisted reproduction, endometrial preparation, estradiol hemihydrate, estradiol treatment, estradiol valerate, frozen embryo transfer, hormone replacement therapy, hrt cycle, vaginal estradiol

## Abstract

Background

Hormone Replacement Therapy (HRT) is a widely used method for endometrial preparation in Frozen Embryo Transfer (FET) cycles. Although multiple estradiol formulations and routes of administration exist, limited data directly compare oral estradiol hemihydrate, vaginal estradiol hemihydrate, and oral estradiol valerate. This study evaluated the efficacy and safety of these three estradiol regimens for endometrial preparation in an assisted reproduction setting.

Objectives

To compare endometrial outcomes, serum estradiol levels, and pregnancy outcomes among three estradiol treatment protocols, oral estradiol hemihydrate, vaginal estradiol hemihydrate, and oral estradiol valerate, in HRT‑based FET cycles.

Methods

This prospective, open‑label, three‑arm investigator‑initiated study was conducted at a single center and included 133 women aged 25-42 years undergoing HRT‑FET cycles. Participants received one of the following regimens: oral estradiol hemihydrate, vaginal estradiol hemihydrate, or oral estradiol valerate. Endometrial thickness, endometrial volume, and serum estradiol levels on progesterone‑start day were measured. Clinical outcomes included serum β-human chorionic gonadotropin (β‑hCG) positivity and clinical pregnancy rates. Data were analyzed using descriptive and comparative statistics.

Results

The mean daily estradiol dose was significantly lower with vaginal estradiol hemihydrate compared to both oral arms (p<0.001). Endometrial thickness and volume showed no significant differences among groups. Mean serum estradiol levels were highest with vaginal estradiol hemihydrate (701.63 pg/mL), significantly exceeding levels with oral estradiol hemihydrate (331.84 pg/mL; p=0.042). Serum β‑hCG positivity rates were 58.33%, 47.62%, and 40.47% (p=0.375) and clinical pregnancy rates were comparable at 44.0%, 28.0%, and 22.7% (p=0.168) in oral estradiol hemihydrate, vaginal estradiol hemihydrate, and oral estradiol valerate groups, respectively.

Conclusions

Estradiol hemihydrate, whether administered orally or vaginally, demonstrated non‑inferior efficacy to oral estradiol valerate for endometrial preparation in FET cycles. Vaginal estradiol hemihydrate achieved adequate endometrial development at significantly lower doses overcoming the barriers of first pass metabolism and minimizing the safety concerns of thrombo-embolism.

## Introduction

Infertility is a growing health problem with as many as 3.7% women affected by infertility globally [1]. About 25% of the world’s infertile couples reside in India [2]. According to mathematical modelling done by Faddy et al., there would be approximately 157 million to 357 million people born through assisted reproductive technology (ART) by the turn of the 21st century [3]. ART has progressed substantially since the landmark report of the first live birth achieved through in‑vitro fertilization (IVF) in 1978 [4]. Successful cryopreservation of human embryos was first reported in 1983 using a slow-cooling technique, leading to the first live birth in 1984 [5]. These developments changed the landscape of IVF treatment, and eventually, the method of rapid cooling and thawing known as vitrification was developed. With continued advancements in embryo cryopreservation, particularly the widespread adoption of vitrification, and the availability of robust safety data, frozen embryo transfer (FET) has become an increasingly utilized component of assisted reproduction [5].

The application of FET has gradually extended beyond its initial use in cases where surplus high-quality embryos remained following elective single embryo transfer during the first treatment cycle. Although elective embryo freezing was initially introduced to protect patients at high risk of ovarian hyperstimulation syndrome [6,7], its role has since expanded. FET is now routinely incorporated into treatment strategies involving pre‑implantation genetic testing, elevated serum progesterone levels detected during the late follicular phase of controlled ovarian stimulation, and clinical scenarios characterized by suboptimal synchrony between embryo development and endometrial receptivity in the fresh transfer cycle. FET has improved the cumulative pregnancy rate for each patient while eliminating multiple pregnancies [8]. It has also improved neonatal outcomes and decreased the risk of ectopic pregnancy [9].

In parallel, treatment protocols employing gonadotropin‑releasing hormone (GnRH) antagonists with GnRH agonist triggering, followed by a comprehensive freeze‑all approach, have gained acceptance in clinical practice. Subsequent embryo transfer in a later FET cycle using this strategy has demonstrated favorable outcomes, including higher live birth rates when compared with FET in select populations [6].

Endometrial preparation remains a critical determinant of success in FET cycles [6], The receptivity of the endometrium and pregnancy outcomes following embryo transfer in the course of IVF cycles are indicated by endometrial thickness. Appropriate endometrial thickness is necessary for successful implantation. Many studies have reported a low rate of pregnancy in cases of thin endometrium [10,11]. Multiple preparation protocols are currently available for endometrial preparation, which include true natural cycles relying on spontaneous ovulation, modified natural cycles utilizing human chorionic gonadotrophin (hCG) for ovulation induction, hormone replacement therapy (HRT) cycles conducted with or without GnRH agonist suppression, and ovarian stimulation cycles using gonadotropins, with or without the addition of letrozole [6]. HRT cycles represented 55% of FET and oocyte donation cycles [8]. 

Using a mouse model, Ma et al. demonstrated that a very narrow range of estrogen levels determined the window of uterine receptivity [12]. Estrogen is responsible for endometrium thickening throughout the follicular phase [13]. Following adequate estrogen primining, progesterone prepares the endometrium for implantation [14]. Therefore, both estrogen and progesterone form a part of the HRT given in FET cycles.

The HRT protocol, initially designed for embryo transfers in recipients of donated eggs [15], has also shown success in the broader population. This has allowed its benefits, such as minimal monitoring and convenient scheduling, to be extended to those undergoing IVF in general. Nevertheless, using HRT cycles universally might have drawbacks, including higher costs, inconvenience, and potential negative effects linked to estrogen supplementation, such as an increased risk of thrombosis [6,16].

Estrogens can be administered through oral, vaginal, and parenteral (transdermal) methods, utilizing both natural and synthetic forms. A meta-analysis determined that neither the type of estrogen supplementation nor the method of administration influenced the success rates of FETs [6].

Women undergoing HRT for FET will often require additional estrogen supplementation, or other intervention, if their endometrium is inadequate (<8 mm) [17]. Estradiol (E2) is often either esterified or micronized; pro-drug esters like estradiol valerate and estradiol acetate are quickly hydrolyzed after absorption, releasing E2 into the bloodstream. Meanwhile, the microcrystalline form of micronized E2, mainly as estradiol hemihydrate, enhances absorption due to its increased surface area, thereby reducing first-pass metabolism [18]. E2 and its hemihydrate form are identical in terms of bioequivalence and activity, with only 3% difference in potency by weight (attributed to presence of water molecules in hemihydrate form). Estradiol hemihydrate is more hydrated than anhydrous estradiol valerate (more insoluble in water), which may result in slower absorption rates with specific formulations of the drug such as vaginal tablets. Administration of 1 mg of oral micronized estradiol hemihydrate resulted in an E2 Cmax of 40-50 pg/mL and an E1 Cmax of 200 pg/mL. Pharmacokinetic parameters with oral administration of 2 mg of micronized estradiol hemihydrate had a Tmax of 8.2 hours and a terminal half-life of 13.5 hours for E2. Estradiol valerate is an ester of the C17-hydroxy group of E2 with valeric acid. This formulation prevents the usual metabolism of E2 to estrone until hydrolysis has taken place. Upon hydrolysis in the intestines to E2 and valeric acid, the resulting E2 is rapidly absorbed [19]. A crossover study done by Wiegratz et al. demonstrated that 2 mg of oral micronized estradiol hemihydrate produced significantly higher serum E2 concentrations during certain intervals than 2 mg of oral estradiol valerate [20].

Generally, 6 mg of E2 is administered orally as either a fixed constant dose (6 mg daily) or in an incremental fashion [15]. According to a study conducted by Banker et al. [19], oral estradiol hemihydrate and estradiol valerate are therapeutically equivalent and provide similar clinical outcomes in an IVF setting. Vaginal E2 can serve as an effective alternative, particularly for patients who exhibit a poor endometrial response to oral E2 [18]. While specially designed creams are available, many professionals opt to administer oral tablets vaginally [18]. Serum levels are significantly elevated, approximately eight times greater than those achieved through oral administration, indicating that this method is exceptionally effective for delivering E2 directly to the target tissue. Moreover, endometrial tissue levels of E2 were observed to be even higher, reaching 80 times the levels seen with oral administration [18,15]. There is limited literature available on the exclusive usage of oral E2 tablets administered vaginally for endometrial preparation in FET cycles.

Against this backdrop, this prospective, open-label, and three‑arm investigator-initiated study was conducted to compare the efficacy and safety of three E2 treatment protocols (estradiol hemihydrate administered orally, estradiol hemihydrate administered vaginally, and estradiol valerate administered orally) for endometrial preparation in FET cycles at our center. The efficacy of three different protocols was measured in terms of endometrial thickness and endometrial volume. Serum E2 levels on progesterone start day, serum β-hCG positivity and clinical pregnancy rates were also measured.

## Materials and methods

Study design

This prospective, open‑label, three‑arm, investigator‑initiated study was conducted to evaluate and compare the efficacy and safety of three E2‑based regimens used for endometrial preparation in FET cycles. Eligible patients were randomized sequentially in a 1:1:1 manner to three treatment arms: oral estradiol hemihydrate (Arm 1), vaginal estradiol hemihydrate (Arm 2), and oral estradiol valerate (Arm 3). Patients who were not comfortable with vaginal treatment were shifted to oral therapy. Patients with previous treatment failure or inadequate response to estradiol valerate were switched to estradiol hemihydrate, while those with prior failure on estradiol hemihydrate were switched to estradiol valerate. The study was conducted at Akanksha Hospital, Anand, Gujarat, India, over a defined period from August 12, 2024 to September 17, 2025. A total of 133 women who underwent FET cycles using an HRT protocol during the study period were enrolled.

Participants were allocated to three treatment arms: arm 1 comprised 29 patients receiving oral estradiol hemihydrate; arm 2 included 57 patients receiving vaginal estradiol hemihydrate; and arm 3 consisted of 47 patients treated with oral estradiol valerate. The embryos transferred in these cycles included frozen-thawed embryos derived from autologous oocytes, donor oocytes, or donor embryos. Accordingly, the embryo source was categorized as thawed self-embryos or thawed donor oocyte/donor embryo (OD/ED) embryos. As this was a prospective study, prior approval was obtained from the institutional ethics committee before participant recruitment. All patients were adequately counseled regarding the treatment protocols, and written informed consent was obtained from each participant for the use and analysis of their treatment data and clinical outcomes before initiation of therapy. Allocation to one of the three treatment arms was performed based on the treating physician’s clinical judgment.

Inclusion and exclusion criteria

Female patients who came for HRT FET cycles, aged 25 to 42 years, and having a normal pelvic ultrasonography report were included in this study.

Women who had a history of smoking, unexplained vaginal bleeding, Asherman’s syndrome, malignancy/HIV/tubercular endometritis, or hypersensitivity of E2 preparations, or any medical conditions that forbid the use of E2 preparations were excluded from the study.

Study protocol

This study compared the efficacy and safety of three different protocols of E2 tablets for endometrial preparation in HRT FET cycles. The primary end points of the study included endometrial thickness and volume on the day of starting the progesterone therapy. Secondary points included serum β‑hCG positivity rate and clinical pregnancy rate.

The HRT regimen was commenced from day two of the menstrual period. Measurement of endometrial thickness was done between the two echogenic borders of endometrium at the midsagittal plane. All the recruited women were administered estradiol hemihdrate orally (arm 1; tablet Endofert H 2 mg, marketed by Intas Pharmaceuticals Ltd., Ahmedabad, Gujarat, India) or estradiol hemihydrate vaginally (arm 2; Tablet Endofert H 2 mg, marketed by Intas Pharmaceuticals Ltd., Ahmedabad, Gujarat, India) or estradiol valerate orally (arm 3; Tablet Progynova 2 mg, manufactured by Bayer Zydus Pharma Pvt. Ltd., Thane, Maharashtra, India ) for endometrial preparation. Women in either groups were assessed on day 10/11 of the menstrual cycle after the initiation of HRT. A transvaginal scan was performed for assessment of endometrial thickness. Adequate endometrial preparation was defined as an endometrial thickness >7mm.

Data collection

Data regarding age, weight and BMI of patient, duration and type of subfertility (primary or secondary), causes of infertility, prior history of IVF treatment cycles, base line investigations, including serum follicle-stimulating hormone (FSH), serum luteinizing hormone (LH), serum E2, serum anti-Müllerian hormone (AMH), serum prolactin and serum thyroid-stimulating hormone (TSH), was measured. Details of the type, route, daily dose and duration of estradiol tablet use, endometrial thickness and volume, and serum E2 levels on the progesterone start day, serum β-hCG positive rate, and clinical pregnancy were also assessed.

Primary end points were endometrial thickness and volume, while secondary end points were serum β-hCG positivity and clinical pregnancy rates.

Statistical analysis

Demographic and baseline characteristics were summarized using descriptive statistics. Categorical variables were summarized with frequency and percentage. Continuous variables were summarized with count, mean, and standard deviation, median, minimum, and maximum. Data from all clinical assessments, whether explicitly referred to in the statistics section or not, were listed and, where appropriate, summarized by categorical information of interest using descriptive statistics. Summary statistics (arithmetic mean, standard deviation, minimum value, maximum value, number of non-missing values) were presented for continuous variables (absolute values at each time point, and if appropriate, changes from baseline) and counts and percentages were presented for categorical variables. Where appropriate the presentation of results were included in shift tables, plots, or statistical tests. Normality of the data was checked by using Shapiro-Wilks test. For comparison between continuous variables, unpaired t-test (for between-group comparison) and ANOVA (for within group comparison), and Chi Square test (for categorical variables) were used. P<0.05 was considered significant. All the analyses were performed using IBM SPSS Statistics for Windows, Version 25 (Released 2020; IBM Corp., Armonk, New York, United States).

Ethical approval

The study was conducted as per the Indian Council of Medical Research (ICMR) Guidelines for Biomedical Research on Human Subjects, the International Council for Harmonization of Technical Requirements for Pharmaceuticals for Human Use - Good Clinical Practices (ICH GCP) Guidelines E6 (R1), Declaration of Helsinki (Fortaleza, Brazil, October 2013) and with other applicable guidelines. The study protocol was reviewed and approved by the Sat Kaival Hospital Pvt. Ltd. Ethics Committee (approval no: SKHPLEC-JUN-2024-001).

## Results

Demographic characteristics

The demographic data are illustrated in Tables 1, 2. There were no significant differences in age and BMI observed between the groups.

**Table 1:** Demographic profile of the study population ANOVA was used to calculate the p value.

Variables	Arm 1: Oral Estradiol hemihydrate (N=29)		Arm 2: Vaginal Estradiol hemihydrate (N=57)		Arm 3: Oral Tablet Estradiol valerate (N=47)		p value
	Mean ± SD	Median (range)	Mean ± SD	Median (range)	Mean ± SD	Median (range)	
Age (years)	33.41±6.14	33.00 (22.00-43.00)	34.09±4.46	34.00 (26.00-44.00)	32.38±4.98	33.00 (21.00-45.00)	0.236
Weight (Kg)	59.07±12.22	58.00 (37.80-95.00)	59.37±13.77	58.00 (36.00-101.00)	62.47±12.26	61.00 (45.00-102.00)	0.395
BMI (Kg/m^2^)	24.49±3.79	25.30 (16.10-34.50)	24.68±4.79	25.40 (16.00-39.50)	25.50±4.64	25.20 (17.80-40.30)	0.558
Duration of infertility (years)	7.25±5.13	5.50 (1.00-19.00)	6.27±9.45	4.00 (1.00-67.00)	4.15±2.78	3.00 (1.00-13.00)	0.127

**Table 2:** Type and causes of infertility and the number of previous in-vitro fertilization (IVF) attempts ANOVA was used to calculate the p value; *Data not captured/missing; PCOS: Polycystic ovarian syndrome.

	Arm 1: Oral Estradiol hemihydrate	Arm 2: Vaginal Estradiol hemihydrate	Arm 3: Oral Estradiol valerate	p value
Type of infertility				
Number of patients (N)	N=28*	N=55*	N=46*	
Primary n (%)	21 (75.0)	35 (63.63)	31 (37.39)	0.579
Secondary n (%)	7 (25.0)	20 (36.37)	15 (32.61)	
Causes of infertility				
Number of patients (N)	N=28*	N=57	N=46*	
Tubal disease; n (%)	5 (17.85)	7 (12.28)	2 (4.34)	0.165
PCOS; n (%)	3 (10.71)	16 (28.07)	7 (15.21)	0.104
Endometriosis; n (%)	1 (3.57)	3 (5.26)	2 (4.34)	0.936
Diminished ovarian reserve; n (%)	9 (32.14)	19 (33.33)	11 (23.91)	0.555
Male factor infertility; n (%)	13 (46.42)	18 (31.57)	18 (39.13)	0.394
Other cause n (%)	0	1 (1.74)	1 (2.17)	0.747
Unexplained infertility; n (%)	5 (17.85)	7 (12.28)	8 (17.39)	0.705
Both male and female factors; n (%)	2 (7.14)	0	1 (2.17)	0.117
Number of previous IVF attempts				
Number of patients (N)	N=28*	N=55*	N=44*	
0; n (%)	23 (82.1)	34(61.8)	30 (68.2)	0.169
1; n (%)	4 (14.3)	11 (20.0)	11 (25.0)	<0.001
2; n (%)	1 (3.6)	6 (10.9)	0	0.053
3; n (%)	0	2 (3.6)	2 (4.5)	0.539
4; n (%)	0	2 (3.6)	0	0.264
5; n (%)	0	0	1 (2.3)	0.386

The baseline investigations (Table 3) showed no statistically significant difference in any of the arms.

**Table 3:** Baseline investigations perfomed on the study population *Data not captured/missing; ANOVA was used to calculate the p values; FSH: Follicle-stimulating hormone; LH: Luteinizing hormone; E2: Estradiol.

Baseline investigations	Arm 1: Oral Estradiol hemihydrate		Arm 2: Vaginal Estradiol hemihydrate		Arm 3: Oral Estradiol valerate		p value
	Mean ± SD	Median (range)	Mean ± SD	Median (range)	Mean ± SD	Median (range)	
Serum anti-müllerian hormone (ng/mL)	n=15*		n=31*		n=31*		0.369
	4.47±5.42	2.17 (0.07-16.98)	5.61±5.25	4.06 (0.05-24.43)	4.06±2.49	3.18 (0.09-12.17)	
Serum prolactin level (ng/mL)	n=12*		n=22*		n=25*		0.234
	24.46±25.28	14.38 (2.91-73.00)	14.39±9.50	11.89 (2.89-44.50)	17.65±15.66	15.00 (6.00-89.30)	
Serum thyroid-stimulating hormone (mIU/L)	n=12*		n=22*		n=18*		0.465
	4.10±5.19	2.21 (0.66-15.00)	3.07±2.88	2.41 (0.01-14.40)	2.62±1.35	2.51 (0.10-6.30)	
Basal serum FSH level (mIU/mL)	n=1*		n=7*		n=9*		0.193
	2.70±0.0	2.70 (2.70-2.70)	4.70±3.78	3.57 (0.78-11.80)	8.35±4.63	7.00 (4.02-17.81)	
Basal serum LH level (mIU/mL)	n=11*		n=23*		n=28*		0.472
	4.93±3.55	4.40 (0.60-12.40	5.97±3.93	4.88 (0.57-16.70)	4.82±3.00	3.91 (0.77-14.85)	
Basal E2 level (pg/mL)	n=10*		n=19*		n=28*		0.816
	117.79±111.64	77.05 (6.90-313.00)	71.91±94.62	30.80 (8.72-329.0)	104.48±277.46	42.50 (3.00-1501.00)	

The details of the estrogen therapy (dosage and duration), endometrial outcomes (endometrial thickness and volume) and pregnancy outcomes (serum β-hCG positivity rate and clinical pregnancy rate) have been mentioned in table 4.

**Table 4:** The frozen embryo transfer (FET) outcomes in the three groups *Data not captured/missing; ANOVA was used to calculate the p values; hCG: human chorionic gonadotropin.

Outcomes	Arm 1: Oral Estradiol hemihydrate	Arm 2: Vaginal Estradiol hemihydrate	Arm 3: Oral Estradiol valerate	p value
Daily dose of estradiol tablet (mg)	5.85 ± 0.770; n=27*	4.12±0.480; n=50*	5.90±0.61; n=42*	<0.001
Duration of estradiol tablet (days)	9.28 ± 2.77; n=25*	8.87±1.70; n=45*	9.00±1.80; n=46*	0.713
Endometrial thickness on the day of starting the progesterone (mm)	12.19±16.59; n=25*	9.22±1.81; n=46*	9.13±1.40; n=46*	0.231
Endometrial volume on the day of starting the progesterone (cc)	3.60±2.27; n=23*	5.22±3.59; n=36*	4.28±3.24; n=32*	0.154
Serum Estradiol levels on the day of starting the progesterone (pg/mL)	331.84±315.26; n=24*	701.63±841.12; n=45*	500.83±1649.86; n=45*	0.440
Serum β-hCG positivity rate	58.33%; 14/24	47.62%; 20/42	40.47%; 17/42	0.375
Clinical pregnancy rate	44.0%; 11/25	28.0%; 14/50	22.7%; 10/44	0.168

While the mean daily dose of E2 was comparable in arm 1 and arm 3 (p=0.754), it was significantly lesser in arm 2 (p<0.001). The mean duration of E2 treatment was comparable in all three arms (p=0.713). The mean endometrial thickness achieved on the starting day of the progesterone was comparable between arm 2 and arm 3 (p=0.798), but was more in arm 1 (12.19 mm). The mean serum E2 level (701.63 pg/mL) on the day of starting of progesterone was more in arm 2 as compared to arms 1 and 3 (p=0.440). 

Subgroup analysis was performed to determine the outcome differences among the arms. On comparing arm 1 with arm 2 (Table 5), it was found that mean daily dose of E2 was significantly higher in arm 1 (5.85±0.77 mg) than in arm 2 (4.12±0.48 mg; p<0.001), while the duration of E2 therapy was nearly similar in both the arms (p=0.442).

**Table 5:** Comparative frozen embryo transfer (FET) outcomes *Data not captured/missing; Unpaired t-test was used to calculate the p values; hCG: human chorionic gonadotropin.

Outcomes	Arm 1: Oral Estradiol hemihydrate	Arm 2: Vaginal Estradiol hemihydrate	p value
Daily dose of estradiol tablet (mg)	5.85 ±0.770; n=27*	4.12±0.480; n=50*	<0.001
Duration of estradiol tablet (days)	9.28±2.77; n=25*	8.87±1.70; n=45*	0.442
Endometrial thickness on the day of starting the progesterone (mm)	12.19±16.59; n=25*	9.22±1.81; n=46*	0.232
Endometrial volume on the day of starting the progesterone (cc)	3.60±2.27; n=23*	5.22±3.59; n=36*	0.059
Serum estradiol levels on the day of starting the progesterone (pg/mL)	331.84±315.26; n=24*	701.63±841.12; n=45*	0.042
Serum β-hCG positivity rate	58.33%; 14/24	47.62%; 20/42	0.402
Clinical pregnancy rate	44.0%; 11/25	28.0%; 14/50	0.165

The mean endometrial thickness and mean endometrial volume were comparable in both arms. Mean serum E2 levels was significantly lower in arm 1 (331.84±315.26 pg/mL) than in arm 2 (701.63±841.12 pg/mL; p=0.042). While serum β-hCG positive rate and clinical pregnancy rate was more in arm 1 (58.33% and 44%, respectively) as compared to arm 2 (47.62% and 28%, respectively), the difference was not significant.

Table 6 compares the FET outcomes in arm 1 and arm 3, where the mean daily dose was the same and the mean duration of E2 therapy, mean endometrial thickness and mean endometrial volume on the day of starting the progesterone were comparable.

**Table 6:** Comparative frozen embryo transfer (FET) outcomes *Data not captured/missing; Unpaired t-test was used to calculate the p values; hCG: human chorionic gonadotropin.

Outcomes	Arm 1: Oral Estradiol hemihydrate	Arm 3: Oral Estradiol valerate	p value
Daily dose of estradiol tablet (mg)	5.85 ±0.770; n=27*	5.90±0.61; n=42*	0.754
Duration of estradiol tablet (days)	9.28±2.77; n=25*	9.00±1.80; n=46*	0.609
Endometrial thickness on the day of starting the progesterone (mm)	12.19±16.59; n=25*	9.13±1.40; n=46*	0.216
Endometrial volume on the day of starting the progesterone (cc)	3.60±2.27; n=23*	4.28±3.24; n=32*	0.396
Serum estradiol levels on the day of starting the progesterone (pg/mL)	331.84±315.26; n=24*	500.83±1649.86; n=45*	0.622
Serum β-hCG positivity rate	58.33%; 14/24	40.41%; 17/42	0.162
Clinical pregnancy rate	44.0%; 11/25	22.72%; 10/44	0.064

While serum β-hCG positivity and clinical pregnancy rates were more in arm 1 (58.33% and 44.0%, respectively) as compared to arm 3 (40.41% and 22.72%, respectively), the difference was not significant.

Table 7 compares the FET outcomes of arm 2 and arm 3.

**Table 7:** Comparative frozen embryo transfer (FET) outcomes *Data not captured/missing; Unpaired t-test was used to calculate the p values; hCG: human chorionic gonadotropin.

Outcomes	Arm 2: Vaginal Estradiol hemihydrate	Arm 3: Oral Estradiol valerate	p value
Daily dose of estradiol tablet (mg)	4.12±0.480; n=50*	5.90±0.61; n=42*	<0.001
Duration of estradiol tablet (days)	8.87±1.70; n=45*	9.00±1.80; n=46*	0.717
Endometrial thickness on the day of starting the progesterone (mm)	9.22±1.81; n=46*	9.13±1.40; n=46*	0.798
Endometrial volume on the day of starting the progesterone (cc)	5.22±3.59; n=36*	4.28±3.24; n=32*	0.263
Serum Estradiol levels on the day of starting the progesterone (pg/mL)	701.63±841.12; n=45*	500.83±1649.86; n=45*	0.469
Serum β-hCG positivity rate	47.62%; 20/42	40.47%; 17/42	0.509
Clinical pregnancy rate	28.0%; 14/50	22.72%; 10/44	0.558

Here the mean daily dose E2 was significantly higher in arm 3 vs arm 2 (p<0.001), while the mean duration of E2 therapy, mean endometrial thickness, and mean endometrial were comparable. The mean serum E2 levels was more in arm 2 (701.63 pg/mL) as compared to arm 3 (500.83 pg/mL), though the difference was not significant. Also, serum β-hCG positivity and clinical pregnancy rates were comparable in both arms.

## Discussion

FET preparation methods can largely be divided into HRT and natural cycles (NCs). Although originally developed to allow embryo transfers in recipients of donated oocytes, the HRT protocol has proven successful in the general population as well, thus extending its advantages in terms of minimal monitoring and easy scheduling to those performing IVF overall [6]. Most HRT protocols empirically opt to supplement estrogens for two weeks in an attempt to mimic the NC [6].

Estrogen supplementation is an essential and indispensable component of all HRT protocols. Estrogens occur naturally in three primary forms: estrone (E1), estradiol (E2), and estriol (E3). Among these, E2, commonly referred to as estradiol‑17β, is the most potent and biologically active estrogen. In clinical practice, oral estrogen preparations are frequently favored due to their ease of use, wide availability, and rapid cessation when required [19].

Estrogen used for therapeutic purposes is available in three principal formulations: E2, ethinylestradiol (EE), and conjugated equine estrogens (CEE). Of these, estradiol‑17β or E2 represents the predominant endogenous estrogen in humans and is marketed in both oral and transdermal forms. However, its oral bioavailability is limited to less than 10%, necessitating pharmaceutical modification. To address this limitation, E2 is commonly administered in esterified or micronized forms. Esterified formulations such as estradiol valerate and estradiol acetate function as prodrugs and are rapidly hydrolyzed after absorption, resulting in the release of active E2 into systemic circulation. Alternatively, micronized E2, most often formulated as estradiol hemihydrate, possesses an increased surface area due to its microcrystalline structure, which enhances intestinal absorption and reduces hepatic first‑pass metabolism [21].

Non‑oral routes of estrogen administration, including intramuscular injection, transdermal delivery, and vaginal application, bypass hepatic first‑pass metabolism. Prolonged hepatic exposure, associated with oral estrogen use, may increase the risk of venous thromboembolic events, particularly among women with predisposing risk factors [18]. Vaginal administration of E2 represents an effective alternative strategy, especially in patients demonstrating inadequate endometrial proliferation with standard estrogen dosing. Although specifically formulated vaginal estrogen products are available, oral E2 tablets are commonly used vaginally in clinical practice [18]. This approach is cost‑effective, easily accessible, and provides sufficient systemic absorption while offering a more targeted effect on the endometrium [18].

In our study, for the group given vaginal estradiol hemihydrate, the mean (SD) daily dose was 4.12 (0.48) mg/day, which was significantly lower (p<0.001) than the group given oral estradiol hemihydrate (5.85 (0.77) mg/day) and oral estradiol valerate (5.90 (0.61) mg/day). This implies that vaginal administration of estradiol hemihydrate requires less daily dose, since it bypasses the hepatic-first pass effect.

In our study, there was no significant difference in mean endometrial thickness (p=0.232) and mean endometrial volume (p=0.059) when oral and vaginal administration of estradiol hemihydrate was compared. This shows that vaginal administration of estradiol hemihydrate is equally effective. Therefore vaginal administration of E2, especially in patients with poor endometrial response to oral E2, can be a valuable alternative. 

When we compared oral administration of estradiol hemihydrate with oral administration of estradiol valerate, there were no significant differences in endometrial thickness (p=0.216) and endometrial volume (p=0.396) on the starting day of progesterone in both the groups. However, a retrospective study by Banker et al. 2021 showed that while the endometrial thickness achieved by both compounds (oral estradiol hemihydrate and estradiol valerate) was adequate, there was a significant increase (of 0.351 mm; p< 0.0001) in thickness in the hemihydrate group [19]. Another recent study comparing the serum E2 levels, according to dose and formulation, in postmenopausal women using HRT found similar serum E2 levels with estradiol valerate and hemihydrate [22]. But another recent study, conducted by Vartanyan et al., reported increased thickness with estradiol hemihydrate as compared to estradiol valerate, though both were administered transdermally [23].

When we compared mean endometrial thickness in those administered vaginal estradiol hemihydrate with those given oral estradiol valerate, there was no significant difference (p=0.798). Similarly, endometrial volume was also not significantly different (p=0.263) between the two groups. This implies that both the formulations were comparable in terms of efficacy.

Mean (SD) serum E2 level in those administered oral estradiol hemihydrate was 331.84 (315.26) pg/mL, which was significantly lower than the group given vaginal estradiol hemihydrate (701.63 (841.12) pg/mL; p=0.042), implying that vaginal administration is a highly efficient method for the delivery of E2.

In our study, mean (SD) serum E2 level in those given vaginal estradiol hemihydrate was 701.63 (841.12) pg/mL, which was not significantly different from those administered oral estradiol valerate (500.83 (1649.86) pg/mL; p=0.469). This suggests that estradiol hemihydrate tablets, when administered vaginally, can be absorbed into the circulation adequately.

For implantation to occur successfully, the endometrium needs to undergo key alterations to receive the growing embryo within a defined period known as the “implantation window”. This complicated process is adjusted by the interaction of adhesion molecules, ovarian hormones, growth factors, and cytokines. Accordingly, one may deduce that vaginal E2 can improve the endometrial development by affecting not only the thickness of the endometrium but also its microenvironment [18].

The initial test done to evaluate the success of implantation is the serum β-hCG level. In our study, serum β-hCG positivity rates were 58.33%, 47.62% and 40.47% in the oral estradiol hemihydrate, vaginal estradiol hemihydrate, and oral estradiol valerate groups, respectively and were comparable (p=0.375).

Similarly, clinical pregnancy rates were 44%, 28% and 22.7% in the oral estradiol hemihydrate, vaginal estradiol hemihydrate, and oral estradiol valerate groups, respectively and were also comparable (p=0.168).

FET remains a cornerstone in ART cycles, and HRT will be an important part of FET protocols. Various preparations and routes for E2 are available for endometrial priming, including oral, vaginal, and transdermal routes. While estradiol valerate has been in use in HRT cycles for a while, in recent times, ART specialists have started using estradiol hemihydrate in HRT FET cycles. Our study showed that the estradiol hemihydrate formulation was non-inferior to the valerate formulation and can also be given via vaginal route to overcome the barriers of first-pass metabolism.

Limitations

We did not assess the vaginal estradiol valerate as it is not practiced at our center. Our study is limited by lack of randomization and conducting the study at multiple centers and geographies. There is a need for larger randomized controlled trials comparing various routes and formulations to elucidate further on this pertinent query of which estradiol formulation or route is ideal for endometrial preparation in HRT FET cycles.

## Conclusions

Based upon the results obtained in our study, it may be suggested that estradiol hemihydrate was comparable to estradiol valerate in terms of achieving adequate endometrial thickness and volume in HRT FET cycles. Also, estradiol hemihydrate, when administered by the vaginal route, was found to be comparable to oral estradiol valerate and estradiol hemihydrate, suggesting that estradiol hemihydrate administration by the vaginal route may be an option for administering estrogen in HRT FET cycles. Vaginal administration of estradiol hemihydrate may help overcome first-pass metabolism, thereby reducing the estrogen dosage needed, probably minimizing the safety concerns regarding thromboembolism.
